# Proteomic Discovery of Plasma Protein Biomarkers and Development of Models Predicting Prognosis of High-Grade Serous Ovarian Carcinoma

**DOI:** 10.1016/j.mcpro.2023.100502

**Published:** 2023-01-17

**Authors:** Se Ik Kim, Suhyun Hwangbo, Kisoon Dan, Hee Seung Kim, Hyun Hoon Chung, Jae-Weon Kim, Noh Hyun Park, Yong-Sang Song, Dohyun Han, Maria Lee

**Affiliations:** 1 Department of Obstetrics and Gynecology, Seoul National University College of Medicine, Seoul, Republic of Korea; 2 Department of Genomic Medicine, Seoul National University Hospital, Seoul, Republic of Korea; 3 Proteomics Core Facility, Biomedical Research Institute, Seoul National University Hospital, Seoul, Republic of Korea; 4 Transdisciplinary Department of Medicine and Advanced Technology, Seoul National University Hospital, Seoul, Republic of Korea; 5 Department of Obstetrics and Gynecology, Seoul National University Hospital, Seoul, Republic of Korea

**Keywords:** ovarian neoplasms, high-grade serous carcinoma, proteomics, enzyme-linked immunosorbent assay, prognosis, aHR, adjusted hazard ratio, AUC, area under the receiver operating characteristic curve, CI, confidence interval, FDR, false discovery rate, FIGO, Federation of Gynecology and Obstetrics, GO, gene ontology, GSN, gelsolin, HGSOC, high-grade serous ovarian carcinoma, iBAQ, intensity-based absolute quantification, IDS, interval debulking surgery, MS, mass spectrometry, NAC, neoadjuvant chemotherapy, OS, overall survival, PARP, poly(ADP-ribose) polymerase, PDS, primary debulking surgery, PFS, progression-free survival, PRMT1, protein arginine methyltransferase 1, SIGLEC14, sialic acid–binding Ig-like lectin 14, SND1, staphylococcal nuclease, tudor domain containing 1, VCAN, versican

## Abstract

Ovarian cancer is one of the most lethal female cancers. For accurate prognosis prediction, this study aimed to investigate novel, blood-based prognostic biomarkers for high-grade serous ovarian carcinoma (HGSOC) using mass spectrometry–based proteomics methods. We conducted label-free liquid chromatography–tandem mass spectrometry using frozen plasma samples obtained from patients with newly diagnosed HGSOC (n = 20). Based on progression-free survival (PFS), the samples were divided into two groups: good (PFS ≥18 months) and poor prognosis groups (PFS <18 months). Proteomic profiles were compared between the two groups. Referring to proteomics data that we previously obtained using frozen cancer tissues from chemotherapy-naïve patients with HGSOC, overlapping protein biomarkers were selected as candidate biomarkers. Biomarkers were validated using an independent set of HGSOC plasma samples (n = 202) *via* enzyme-linked immunosorbent assay (ELISA). To construct models predicting the 18-month PFS rate, we performed stepwise selection based on the area under the receiver operating characteristic curve (AUC) with 5-fold cross-validation. Analysis of differentially expressed proteins in plasma samples revealed that 35 and 61 proteins were upregulated in the good and poor prognosis groups, respectively. Through hierarchical clustering and bioinformatic analyses, GSN, VCAN, SND1, SIGLEC14, CD163, and PRMT1 were selected as candidate biomarkers and were subjected to ELISA. In multivariate analysis, plasma GSN was identified as an independent poor prognostic biomarker for PFS (adjusted hazard ratio, 1.556; 95% confidence interval, 1.073–2.256; *p* = 0.020). By combining clinical factors and ELISA results, we constructed several models to predict the 18-month PFS rate. A model consisting of four predictors (FIGO stage, residual tumor after surgery, and plasma levels of GSN and VCAN) showed the best predictive performance (mean validated AUC, 0.779). The newly developed model was converted to a nomogram for clinical use. Our study results provided insights into protein biomarkers, which might offer clues for developing therapeutic targets.

## Highlights

• We aimed to investigate novel, blood-based prognostic biomarkers in HGSOC.
• MS-based label-free quantification was conducted using frozen plasma samples.
• Candidate biomarkers were validated with an independent set of samples *via* ELISA.
• Plasma GSN was identified as an independent poor prognostic biomarker for PFS.
• We successfully developed models predicting the 18-month PFS rate for clinical use.

## Graphical Abstract

**Figure:**
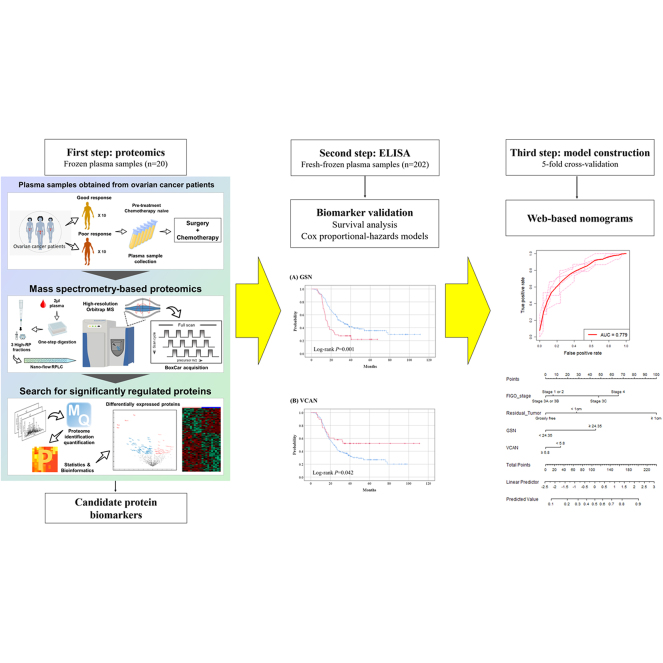

## In Brief

To investigate novel, prognostic protein biomarkers, we conducted label-free liquid chromatography–tandem mass spectrometry using frozen plasma samples obtained from patients with newly diagnosed high-grade serous ovarian carcinoma. Candidate biomarkers underwent validation with an independent set of plasma samples *via* ELISA. By combining clinical factors and ELISA results, we successfully developed models and nomograms to predict the 18-month progression-free survival rate for clinical use.

Ovarian cancer is one of the most lethal cancers among women. Annually, 313,959 new ovarian cancer cases and 207,252 related deaths are expected worldwide (1). The absence of disease-specific early symptoms and effective screening methods leads to ovarian cancer being diagnosed at an advanced stage and having high recurrence and mortality rates despite treatment, consisting of extensive cytoreductive surgery followed by taxane- and platinum-based chemotherapy (2, 3, 4). Meanwhile, ovarian cancer is not a single disease but a heterogeneous disease comprising various histologic subtypes with different carcinogenic routes and clinical features. Among the subtypes of ovarian cancer, high-grade serous ovarian carcinoma (HGSOC) is the most common and responds very well to chemotherapy; however, it frequently relapses, with acquisition of chemoresistance (4).

Since The Cancer Genomic Atlas reported results from integrated genomic analyses of HGSOC (5), the management of HGSOC rapidly evolved. Maintenance therapy with poly(ADP-ribose) polymerase (PARP) inhibitors, such as olaparib and niraparib, was incorporated into the primary treatment of HGSOC based on landmark phase III randomized controlled trials (6, 7, 8). After a complete or partial response to first-line platinum-based chemotherapy, olaparib maintenance therapy can be offered to patients with *BRCA1/2* mutated, advanced HGSOC to improve survival outcomes, while niraparib maintenance therapy confers survival benefits in advanced HGSOC, regardless of *BRCA1/2* mutational status or homologous recombination deficiency. Accurate prediction of prognosis is necessary to facilitate molecular profiling–based HGSOC treatment.

In this regard, our research team has focused on discovering prognostic protein biomarkers in HGSOC using mass spectrometry (MS) proteomics. This emerging technology allows high-throughput and individualized characterization and quantification of proteins in biospecimens (9). Previously, we identified six protein biomarkers associated with progression-free survival (PFS) through the label-free quantitative proteomic analysis of frozen primary HGSOC tissues and validated them using immunohistochemical staining in an independent sample set (10).

However, liquid biopsy has many advantages, such as noninvasiveness, swiftness, real-time monitoring, and the possibility of overcoming tumor heterogeneity (11, 12). Thus, we aimed to investigate whether we could identify novel, prognostic protein biomarkers for HGSOC from blood samples using MS-based proteomics. Biomarker candidates were validated using an enzyme-linked immunosorbent assay (ELISA) in an independent dataset. We also developed models to predict 18-month PFS rates in patients with HGSOC.

## Experimental Procedures

### Ethics Statement

This study was approved by the Institutional Review Board of Seoul National University Hospital (SNUH; No. H-2010-152-1167) and was conducted in accordance with the Declaration of Helsinki. At our institution, we routinely asked patients with newly diagnosed ovarian cancer who were scheduled to undergo primary treatment to donate their biospecimens (*e.g.*, blood, urine, and cancer tissues) for research purposes with written informed consent since June 2012.

### Sample Collection

In this study, we used plasma samples from HGSOC patients that were obtained 1 day before primary debulking surgery (PDS) or initiation of neoadjuvant chemotherapy (NAC) and stored at the Seoul National University Hospital Hunan Biobank. The process for the collection of plasma from whole blood was as follows: Collect 6 ml of blood sample into the EDTA tube, and centrifuge for 10 min at 1551*g* at 4 °C. After centrifugation, carefully collect the plasma layer with a transfer pipette without disturbing the buffy coat layer. Pipette 700 μl of plasma into a 1.8-ml labeled cryovial, up to four vials. Place all aliquots upright in a labeled rack in a −196 °C LN2 tank. All the plasma samples used in this study had never been thawed before.

### Experimental Design and Statistical Rationale

This study included three phases: (1) biomarker discovery through proteomic and bioinformatic analyses, (2) prognostic validation of candidate biomarkers using ELISA, and (3) construction of models predicting the 18-month PFS rate in patients with HGSOC (supplemental Fig. S1).

For the first phase (discovery), we retrieved the frozen plasma obtained from patients who met the following criteria: (1) newly diagnosed with HGSOC between June 2012 and December 2016, without any history or evidence of other malignancies; (2) completed primary treatment, consisting of primary debulking surgery (PDS; not NAC–interval debulking surgery [IDS]) and taxane- and platinum-based adjuvant chemotherapy; and (3) patients whose disease relapsed within 18 months after PDS, that is, PFS <18 months (poor prognosis group) or those whose disease did not relapse for at least 18 months after PDS, that is, ≥18 months of PFS (good prognosis group). Twenty patients from the two groups (10 in each group) were selected for further proteomic analyses. The order of sample preparation was randomized and independent of the patient list. The proteomic profiles of the two groups were compared.

In the second phase (validation), we retrieved pretreatment frozen plasma of patients who met the following conditions: (1) newly administered HGSOC between June 2012 and December 2019, without any history or evidence of other malignancies; (2) completed primary treatment, consisting of either PDS or NAC-IDS, followed by postoperative taxane- and platinum-based adjuvant chemotherapy. We excluded patients if they had enrolled in clinical trials for primary treatment; did not provide written informed consent; or were lost to follow-up during primary treatment or within 18 months after initiation of primary treatment, without relapse or disease progression. A total of 202 consecutive patients with HGSOC were included in this phase, and the sample size was adequate for multivariate survival analysis and further development of predictive models. The order of sample preparation was also randomized and independent of the patient list. ELISA was conducted with technical triplicates on pooling samples for the standard curve and batch control.

In the medical record review, we collected patients’ clinicopathologic data. Disease progression was ascertained based on computed tomography scans by applying the Response Evaluation Criteria in Solid Tumors version 1.1 (13). PFS and overall survival (OS) were defined as the time intervals from the date of initial diagnosis to the date of disease progression and to the date of cancer-related death or last follow-up, respectively.

### Proteomic and Bioinformatic Analyses

**Fig. 1:**
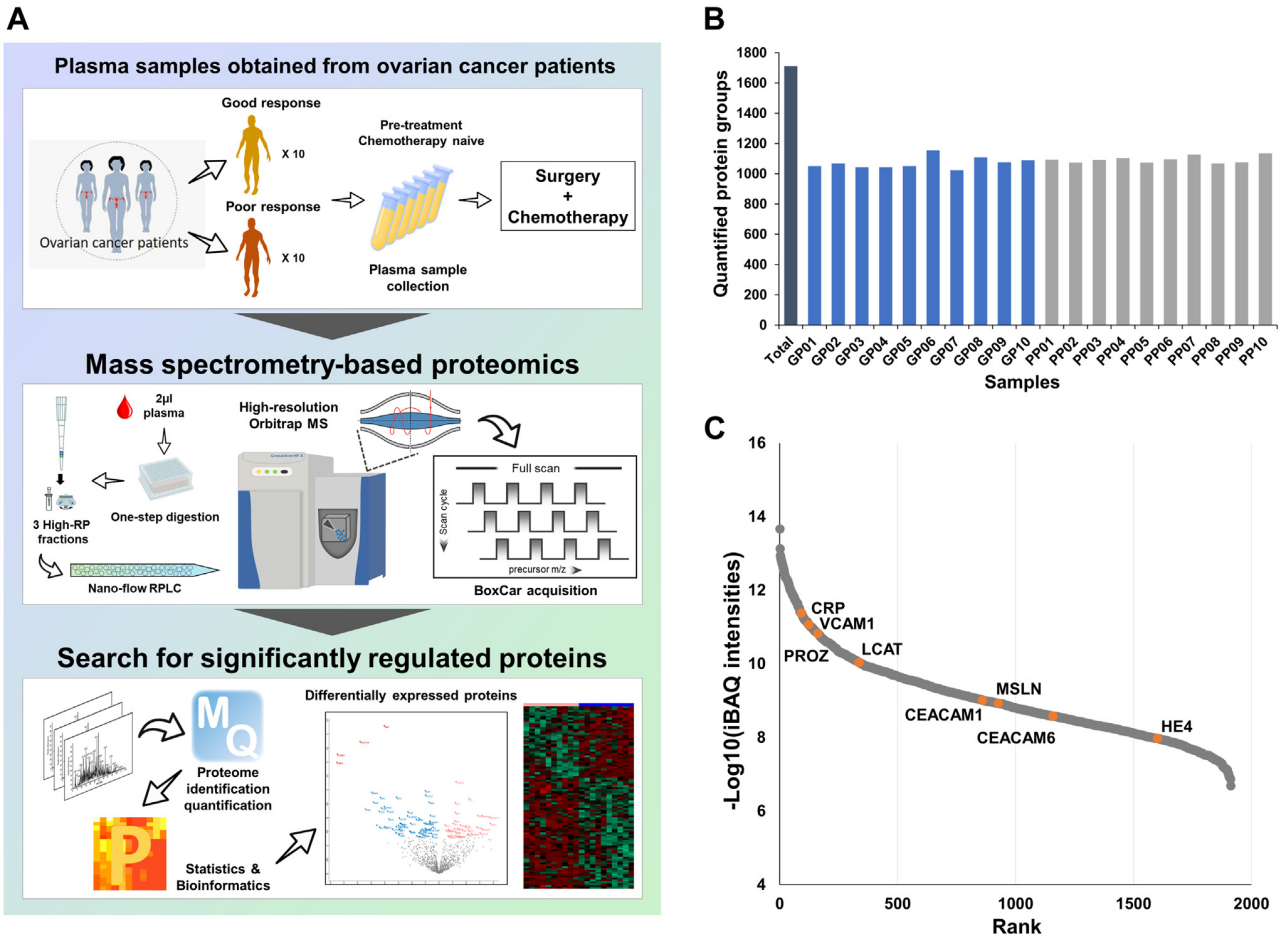
**Proteomic analysis of ovarian cancer blood samples with respect to survival outcome.***A*, overall workflow of proteomic analysis; *B*, total number of proteins identified in each sample; *C*, dynamic range of proteins quantified in our study. Well-known ovarian cancer marker candidates are color coded.

The overall workflow of proteomic and bioinformatic analyses are depicted in Figure 1*A*.

#### Sample Preparation

Protein digestion was performed using 2 μl of each plasma sample as previously described, with some modifications (14, 15). Briefly, 23 μl of protein digestion buffer, including reduction and alkylation reagents, was added to 2 μl plasma samples in 96-well plates. The mixture was boiled for 25 min at 60 °C to denature and alkylate the proteins. After cooling samples to room temperature, protein digestion was performed at 37 °C overnight using a trypsin/LysC mixture (Promega) at a 100:1 protein-to-protease ratio. The second digestion was performed at 37 °C for 2 h using trypsin (enzyme-to-substrate ratio [w/w], 1:1000). All resulting peptides were acidified with 10% trifluoroacetic acid (TFA). The acidified peptides were loaded onto custom-made styrene divinylbenzene reversed-phase sulfonate-StageTips according to previously described procedures (15, 16). The StageTip was washed three times with 100 μl 0.2% TFA. Three fractionations were performed using elution buffers with a step gradient of increasing acetonitrile (40%, 60%, and 80%) in 1% ammonium hydroxide. All the eluted peptides were dried using a SpeedVac centrifuge (Thermo Fisher Scientific).

#### Liquid Chromatography With Tandem MS Analysis

All liquid chromatography with tandem MS (MS/MS) analyses were conducted using an Ultimate 3000 UHPLC system (Dionex) coupled with a Q-Exactive HF-X mass spectrometer (Thermo Fisher Scientific), as previously described, with some modifications (17). Peptides were separated on a two-column system equipped with a trap column (Thermo Fisher Scientific, Acclaim PepMap, C18 5 μm, 100 Å, 300 μm I.D. × 5 mm) and an analytical column (Thermo Fisher Scientific, EASY-Spray column, C18 1.9 μm, 100 Å, 75 μm I.D. × 50 cm) using 90-min gradients from 7% to 30% acetonitrile at a flow rate of 300 nl/min. Column temperature was maintained at 60 °C using a column heater. MaxQuant.Live version 1.2 was used for BoxCar acquisition (18). The MS1 resolution was set to 120,000 at *m/z* 200 for BoxCar, and the acquisition cycle comprised two BoxCar scans at 12 boxes (scaled width, 1 Th overlap) with a maximum ion injection time of 20.8 per box, with the individual AGC target set to 250,000. MS/MS spectra were acquired at a higher-energy collisional dissociation-normalized collision energy of 30, with a resolution of 17,500 at *m/z* 200. The maximum ion injection durations for the full and MS/MS scans were 20 ms and 100 ms, respectively.

#### Data Processing

All raw MS files were processed using MaxQuant (version 1.6.1.0) (19). MS/MS spectra were searched against the Human UniprotKB protein sequence database (December 2014, with 88,657 entries of 20,459 human genes) using the Andromeda search engine (20). Primary searches were performed using 6 ppm precursor ion tolerance for total protein-level analysis. MS/MS ion tolerance was set at 20 ppm. Cysteine carbamidomethylation was used as a fixed modification. Protein N-acetylation and methionine oxidation are considered variable modifications. Enzyme specificity was set to full tryptic digestion. Peptides with a minimum length of six amino acids and up to two missed cleavages were considered. The required false discovery rate (FDR) was set to 1% at peptide, protein, and modification levels. To maximize the number of quantification events across samples, we enabled the “Match between Runs” options on the MaxQuant platform. The MS proteomics data have been deposited to the ProteomeXchange Consortium *via* the PRIDE (21) partner repository with the dataset identifier PXD034646. Annotated MS/MS spectra can be accessed through MS-Viewer (22) (https://msviewer.ucsf.edu/cgi-bin/mssearch.cgi?report_title=MS-Viewer&search_key=bzgazjrsgb&search_name=msviewer) with the following search keys: bzgazjrsgb.

#### Label-Free Quantification and Statistical Analysis

For label-free quantification, the intensity-based absolute quantification (iBAQ) algorithm (23) was used on the MaxQuant platform. Briefly, iBAQ values, determined using MaxQuant, are the raw intensities divided by the number of theoretical peptides (23). Thus, the iBAQ values were proportional to the molar quantities of the proteins. Perseus software was used for statistical analysis (24). First, we eliminated proteins identified as “reverse” and “only identified by site.” After filtering values of at least 70% in each group, missing values were imputed by random numbers drawn from a normal distribution with a width of 0.3 and a down-shift of 1.8. Finally, data were normalized using a width-adjustment function that subtracts the medians and scales all values in a sample to yield equal interquartile ranges (25). For pairwise proteome comparisons, we performed a two-sided *t* test with a significance level (*p* value) of <0.05 and a fold-change of >1.5. Support vector machine analysis was performed using the R/Bioconductor package “GNC” (26).

#### Bioinformatic Analysis

Principal component analysis was performed using Perseus software with proteomic expression profiles (24). Gene ontology (GO) enrichment analysis was performed using the EnrichR analysis tool (https://maayanlab.cloud/Enrichr/), according to the biological process in the GO analysis (27). EnrichR uses the Fisher exact test to calculate *p* values. Statistical significance was set at *p* value <0.05, and GO analysis was used to identify significant GO biological process terms.

### Enzyme-Linked Immunosorbent Assay

ELISA kits for human proteins were used to quantify the plasma levels of endogenous proteins, according to the manufacturer’s instructions. ELISA kits for gelsolin (GSN; abx253831), versican (VCAN; abx153474), staphylococcal nuclease, tudor domain containing 1 (SND1; abx383338), sialic acid–binding Ig-like lectin 14 (SIGLEC14; abx545882), and protein arginine methyltransferase 1 (PRMT1; abx258982) were purchased from Abbexa, whereas a kit for CD163 (DC1630) was purchased from R&D Systems.

After determining the optimal dilution factor for each protein, the concentrations of GSN, VCAN, SND1, CD163, SIGLEC14, and PRMT1 were measured and quantified in the pretreatment frozen plasma samples (n = 202). Absorbance at 450 nm was measured using a SPARK multimode microplate reader (Tecan Systems, Inc).

### Model Construction

We constructed regression-based models to predict 18-month PFS rates using clinical variables and the ELISA results for protein biomarkers in patients with HGSOC (n = 202). The 18-month PFS rate was defined by binarizing the PFS for 18 months. Each of the six identified protein biomarkers was binarized based on the optimal cutoff obtained from maximally selected log-rank statistics (maxstat) (28). To select important predictors for the 18-month PFS rate, stepwise selection was performed based on the area under the receiver operating characteristic curve (AUC). During stepwise selection, predictors contributing to AUC improvement were selected in a stepwise fashion (29). From variable selection to model evaluation, 5-fold cross-validation was used, considering the two-class proportions of the 18-month PFS rate. The AUC, sensitivity, and specificity were used as evaluation measures. The optimal cutoff for calculating sensitivity and specificity was determined as a value corresponding to the maximum value of balanced accuracy, defined as the average of the sensitivity and specificity. Based on the logistic regression model including the selected predictors, we developed a nomogram for clinical use.

R statistical software (version 4.0.3; R Foundation for Statistical Computing) was used to construct predictive models and plot nomograms.

### Statistical Analysis

Clinicopathologic characteristics were compared between the good and poor prognosis groups by using Student’s *t* and Mann–Whitney U tests for continuous variables and Pearson’s chi-squared and Fisher’s exact tests for categorical variables. The Pearson’s correlation coefficient test was used to measure the relationship between continuous variables. For survival analysis, we used the Kaplan–Meier method with the log-rank test. In the multivariate analysis, a Cox proportional hazards model was constructed and adjusted hazard ratios (aHRs) and 95% confidence intervals (CIs) were calculated.

Statistical analyses were performed using SPSS Statistics (version 25.0; IBM Corp) and GraphPad Prism 5 (GraphPad Inc). All statistical tests were two sided, and a *p* value <0.05 was considered statistically significant.

## Results

### Characteristics of Patients in the Discovery Phase

The clinicopathologic characteristics of 20 patients with HGSOC for whom proteomic analysis was performed are presented in supplemental Table S1. The mean patient age was 54.9 years, which was similar between the good and poor prognosis groups (*p* = 0.609). Between the two groups, there was no differences in parity, menopausal status, initial serum CA-125 levels, International Federation of Gynecology and Obstetrics (FIGO) stage, residual tumor after PDS, and total number of cycles of postoperative adjuvant chemotherapy (supplemental Table S2). In relation to germline *BRCA* mutational status, 7 and 2 patients had *BRCA1* and *BRCA2* mutations, respectively, while the other 11 patients harbored wildtype *BRCA1/2*. None of the patients received first-line PARP inhibitor maintenance therapy. The median length of observations was 34.0 months, during which 15 patients experienced disease recurrence. Patients in the good prognosis group had a significantly better PFS than those in the poor prognosis group (median, 48.4 *versus* 12.4 months; *p* < 0.001).

### Results of Proteomic and Bioinformatic Analyses

#### Global Proteomic Analysis of Plasma Samples

To identify prognostic biomarkers for HGSOC, we performed MS-based label-free quantification using frozen plasma samples from chemotherapy-naïve patients (n = 20). To increase the proteome depth, we applied BoxCar acquisition using a small amount (2 μl) of plasma sample, without depletion of highly abundant proteins. In total, 1912 proteins were identified at the protein FDR 1% level. An average of 1082 protein groups were quantified per sample (Fig. 1*B*). Signal intensities for the quantified proteins overall spanned approximately seven orders of magnitude (Fig. 1*C*) and included several previously reported ovarian cancer marker candidates, such as HE4, MSLN, VCAM-1, CEA, CRP, PROZ, LCAT, and M-CSF. Details of the identified and quantified proteins are presented in supplemental Table S3.

To identify the differences within and between groups, the protein profiles were plotted as multiscatter plots. Pearson's correlation coefficient values for proteome pairs were calculated (supplemental Fig. S2). The intragroup correlation displayed average Pearson's correlation coefficient values of 0.84 and 0.83 in the good and poor response groups, respectively. The average intergroup Pearson's correlation coefficient value, between the good and poor response group, was 0.82.

#### Label-Free Quantification

**Fig. 2:**
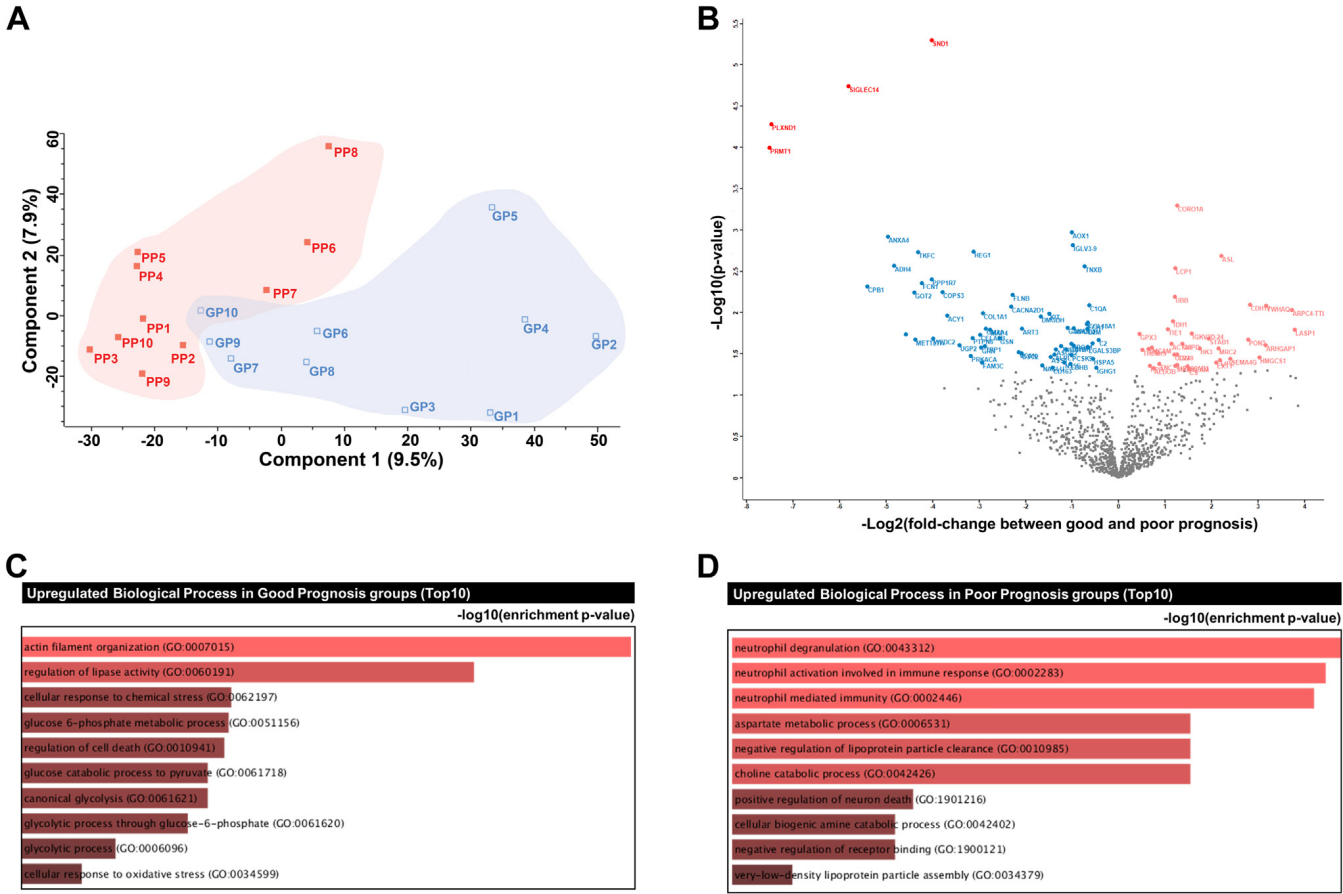
**Statistical and functional differences between good and poor prognosis groups.***A*, principal component analysis; *B*, volcano plot; *C*, gene ontology (GO) biological process enrichment tree-map of upregulated proteins in the good prognosis group; *D*, GO biological process enrichment tree-map of upregulated proteins in the poor prognosis group.

Next, we assessed significant quantitative differences between samples from patients with good and poor prognosis, based on pairwise comparisons. First, we compared the good and poor prognosis groups *via* principal component analysis of a filtered list with approximately 1028 proteins (with 70% valid iBAQ values in at least one group). Although tumor proteomes were correlated regardless of prognosis (supplemental Fig. S2), the two good and poor response groups were separated independently (Fig. 2*A*).

Pairwise comparisons *via t* test and filtering (*p* < 0.05; fold-change, >1.5) revealed significant alterations in 96 proteins, of which 35 proteins had higher expression in the good prognosis group than the poor prognosis group. The other 61 proteins had higher expression in the poor prognosis group than the good prognosis group (Fig. 2*B* and supplemental Table S4). Using the stringent filtering criterion of FDR <0.05, PLXND1, SIGLEC14, SND1, and PRMT1 were found to be upregulated in patients with a poor prognosis. GO enrichment analysis based on biological processes revealed that proteins upregulated in the good prognosis group were significantly enriched for terms such as “actin filament organization,” “regulation of lipase activity,” “cellular response to chemical stress,” “glucose 6-phosphate metabolic process,” and “regulation of cell death” (Fig. 2*C* and supplemental Table S5). In contrast, proteins upregulated in the poor prognosis group were significantly enriched in “neutrophil degranulation,” “neutrophil-mediated immunity,” “aspartate metabolic process,” and “negative regulation of lipoprotein particle clearance” GO-BPs (Fig. 2*D* and supplemental Table S5).

#### Selection of Candidate Prognostic Biomarkers

Potential plasma biomarker candidates for sequential validation experiments were first selected among differentially expressed proteins that met one or more of the following criteria: (1) identified as differentially expressed proteins (PLXND1, SND1, SIGLEC14, and PRMT1) with FDR-adjusted *p* value <0.05 and (2) previously found to be differentially expressed in frozen tissues between the good and poor prognosis groups of patients with HGSOC (10), considering that increased levels of cancer tissue-specific proteins can be released into the blood (30). Consequently, we first selected 18 potential biomarkers (GSN, VCAN, SND1, SIGLEC14, CD163, PRMT1, PLXND1, F12, HPR, HSPA5, ACY1, CD248, C5, GRHPR, MCAM, PPP1R7, STAB1, and UGGT1). Among the 14 proteins that overlapped with our previous tissue data, 6 proteins (GSN, VCAN, CD163, F12, HPR, and HSPA5) were selected according to concordant expression patterns between tissue and plasma. We further selected prognostic biomarker candidates on the basis of the following parameters: (1) the targeted proteins were upregulated in patients with a poor prognosis (upregulated proteins are more suitable as biomarkers than downregulated proteins), (2) a commercial ELISA kit was available for the protein, and (3) proteins could be detected in our validation cohort using the selected ELISA kits. Finally, GSN, VCAN, SND1, SIGLEC14, CD163, and PRMT1 were selected as candidate biomarkers for the validation stage (supplemental Fig. S3).

#### Validation of Protein Biomarkers Through ELISA

Protein biomarkers underwent prognostic validation by using independent plasma samples obtained from patients with HGSOC (n = 202). Clinicopathologic characteristics of the patients are presented in Table 1. Of all patients, 88.6% had advanced-stage (FIGO stage III–IV) disease and 92.1% underwent PDS, rather than NAC followed by IDS. Optimal debulking (with no gross residual tumor) was achieved in 71.8% of cases. Germline and/or somatic *BRCA1/2* testing was conducted in 158 patients (78.2%), and 36.1% (57/158) had mutations in *BRCA1* or *BRCA2*. Three patients received first-line PARP inhibitor maintenance therapy (olaparib). The median length of observation was 43.8 months, during which 134 patients (66.3%) experienced relapse and 30 (14.9%) died of the disease. The median PFS was 24.6 months, and the 18-month PFS rate was 62.9% (127/202) (supplemental Fig. S4).

**Table 1:** Clinicopathologic characteristics of the patients in validation phase

Characteristics	All (n = 202, %)	Good prognosis (n = 127, %)	Poor prognosis (n=75, %)	*P*
Age, years				
Mean ± SD	57.0 ± 10.8	55.8 ± 10.4	59.2 ± 11.2	0.029
Parity				
Median (range)	2 (0–8)	2 (0–6)	2 (0–8)	0.008
Menopausal status				
Menopause	142 (70.3)	85 (66.9)	57 (76.0)	0.173
Serum CA-125, IU/mL				
Median (range)	800.5 (5.1–10000)	654.0 (5.1–10000)	1459.0 (19.5–10000)	0.028
FIGO stage				0.001
I–II	23 (11.4)	20 (15.7)	3 (4.0)	
III	131 (64.9)	86 (67.7)	45 (60.0)	
IV	48 (23.8)	21 (16.5)	27 (36.0)	
Primary treatment strategy				0.267
PDS	186 (92.1)	119 (93.7)	67 (89.3)	
NAC-IDS	16 (7.9)	8 (6.3)	8 (10.7)	
Residual tumor after surgery				<0.001
No gross	145 (71.8)	106 (83.5)	39 (52.0)	
<1 cm	33 (16.3)	17 (13.4)	16 (21.3)	
≥1 cm	24 (11.9)	4 (3.1)	20 (26.7)	
Chemotherapy regimen				0.027
Paclitaxel-Carboplatin	184 (91.1)	120 (94.5)	64 (85.3)	
Paclitaxel-Carboplatin-BEV	18 (8.9)	7 (5.5)	11 (14.7)	
Total cycles of chemotherapy				0.089
4–6	168 (83.2)	110 (86.6)	58 (77.3)	
7–9	34 (16.8)	17 (13.4)	17 (22.7)	
Recurrence				
No	68 (33.7)	68 (53.5)	0	<0.001
Yes	134 (66.3)	59 (46.5)	75 (100.0)	
PSR^a^	104 (77.6)	59 (46.5)	45 (60.0)	<0.001
PRR	30 (22.4)	0	30 (40.0)	
Platinum sensitivity				<0.001
Platinum sensitive^b^	172 (85.1)	127 (100.0)	45 (60.0)	
Platinum resistant	30 (14.9)	0	30 (40.0)	
g/t*BRCA* mutational status^c^				
Not tested (unknown)	44 (21.8)	25 (19.7)	19 (25.3)	0.347
Tested	158 (78.2)	102 (80.3)	56 (74.7)	
Both wildtype	101 (50.0)	57 (44.9)	44 (58.7)	0.017
*BRCA1* mutation	40 (19.8)	31 (24.4)	9 (12.0)	
*BRCA2* mutation	17 (8.4)	14 (11.0)	3 (4.0)	

Abbreviations: BEV, bevacizumab; CA-125, cancer antigen 125; FIGO, International Federation of Gynecology and Obstetrics; IDS, interval debulking surgery; NAC, neoadjuvant chemotherapy; PDS, primary debulking surgery; PRR, platinum-resistant recurrence; PSR, platinum-sensitive recurrence; SD, standard deviation.

a PSR was defined as relapse ≥6 months after completion of taxane- and platinum-based chemotherapy, whereas PRR as relapse <6 months.

b In addition to PSR, the patients who completed taxane- and platinum-based chemotherapy and did not experience disease recurrence during at least 6 months of follow-up period were considered platinum-sensitive.

c Germline and/or somatic *BRCA1/2* mutational status.

Table 1 also compares clinicopathologic characteristics between the good and poor prognosis groups. Patients in the poor prognosis group (n = 75) were significantly older (*p* = 0.029) and had more advanced disease (*p* = 0.001), compared with those in the good prognosis group (n = 127). While the two groups had a similar proportion of PDS (*p* = 0.267), optimal debulking was less frequently achieved in the poor prognosis group (52.0% *versus* 83.5%; *p* < 0.001). Among the patients who received germline and/or somatic *BRCA1/2* testing, *BRCA1/2* mutations were less frequently observed in the poor prognosis group (21.4% *versus* 44.1%; *p* = 0.004). Comparing the survival outcomes, the poor prognosis group showed worse PFS (median, 12.5 *versus* 54.1 months; *p* < 0.001) and OS (5-year OS rate, 57.1% *versus* 94.0%; *p* < 0.001), compared with the good prognosis group (supplemental Fig. S4).

Six protein biomarkers, GSN, VCAN, SND1, SIGLEC14, CD163, and PRMT1, were subjected to further prognostic validation using ELISA (supplemental Table S6). The ELISA results are summarized in Table 2 and supplemental Fig. S5. Table 2 also compares ELISA results between the good and poor prognosis groups. Plasma GSN levels were significantly higher in the poor prognosis group than those in the good prognosis group (median, 23.150 *versus* 19.300 ng/ml; *p* = 0.001). However, plasma levels of VCAN, SND1, SIGLEC14, CD163, and PRMT1 were similar between the two groups.

**Table 2:** ELISA results of the six plasma protein biomarkers

Protein	All (n=202, %)	Good prognosis (n = 127, %)	Poor prognosis (n = 75, %)	*P*
GSN, ng/mL				0.001
Median (range)	20.525 (8.300–96.200)	19.300 (8.300–96.200)	23.150 (11.000–62.950)	
Mean ± SD	22.462 ± 10.095	21.197 ± 10.344	24.603 ± 9.341	
Cutoff	24.350			
VCAN, ng/mL				0.679
Median (range)	4.339 (1.676–15.041)	4.218 (1.687–10.513)	4.408 (1.676–15.041)	
Mean ± SD	4.571 ± 1.754	4.537 ± 1.676	4.629 ±1.888	
Cutoff	5.832			
SND1, ng/mL				0.193
Median (range)	17.670 (1.800–45.040)	17.700 (1.800–45.040)	17.120 (6.160–38.020)	
Mean ± SD	18.440 ± 7.276	19.089 ± 7.733	17.342 ± 6.326	
Cutoff	25.900			
SIGLEC14,^a^ ng/mL				0.315
Median (range)	5.250 (2.700–29.070)	5.125 (2.720–29.070)	5.440 (2.700–20.010)	
Mean ± SD	5.941 ± 3.082	5.828 ± 3.036	6.129 ± 3.169	
Cutoff	6.300			
CD163, ug/uL				0.339
Median (range)	0.583 (0.000–1.608)	0.576 (0.240–1.608)	0.608 (0.000–1.511)	
Mean ± SD	0.643 ± 0.263	0.635 ± 0.255	0.655 ± 0.277	
Cutoff	0.394			
PRMT1, ng/mL				0.947
Median (range)	2.496 (0.816–12.520)	2.456 (0.816–12.520)	2.640 (1.056–6.016)	
Mean ± SD	3.043 ± 1.800	3.184 ± 2.090	2.804 ± 1.126	
Cutoff	4.840			

Abbreviation: SD, standard deviation.

Missing data: ^a^1.

No correlation was observed between serum CA-125 levels and the plasma levels of each protein biomarker (supplemental Table S7). Plasma GSN levels were significantly correlated with plasma VCAN (Pearson’s correlation coefficient *r* = 0.224; *p* = 0.001), SND1 (*r* = 0.177; *p* = 0.012), and CD163 levels (*r* = 0.351; *p* < 0.001), but the correlations were weak. A weak positive correlation was also observed between plasma VCAN and SND1 levels (*r* = 0.167; *p* = 0.017). Plasma VCAN levels were moderately correlated with plasma SIGLEC14 levels (*r* = 0.501; *p* < 0.001) and weakly correlated with plasma CD163 levels (*r* = 0.341; *p* < 0.001). Using the cutoff values determined by maxstat (28), the validation set was divided into high (≥cutoff value) and low (<cutoff value) plasma level groups for each protein.

We then compared the clinicopathologic characteristics of the patients with high and low plasma levels of the six protein biomarkers (supplemental Table S8). Patients with high GSN levels (n = 62) were significantly older (*p* = 0.001), had higher initial serum CA-125 levels (*p* = 0.043), had more advanced disease (*p* = 0.012), less commonly achieved optimal debulking (*p* = 0.011), and more commonly showed platinum resistance (*p* = 0.040) than did those with low GSN levels (n = 140). For VCAN, high plasma levels were associated with old age at the initial diagnosis (*p* < 0.001). For SND1, high plasma levels were associated with advanced disease (*p* = 0.032) and suboptimal debulking (*p* = 0.027). However, for SIGLEC14, CD163, and PRMT1, no significant differences in patient age, FIGO stage, or residual tumor after surgery were observed between the high and low expression groups.

In assessing the platinum sensitivity of patients with respect to the plasma levels of each protein biomarker, we observed a significant difference only for GSN. Patients with high GSN levels were less sensitive to platinum-based chemotherapy than those with low GSN levels (77.4% *versus* 88.6%; *p* = 0.040).

**Fig. 3:**
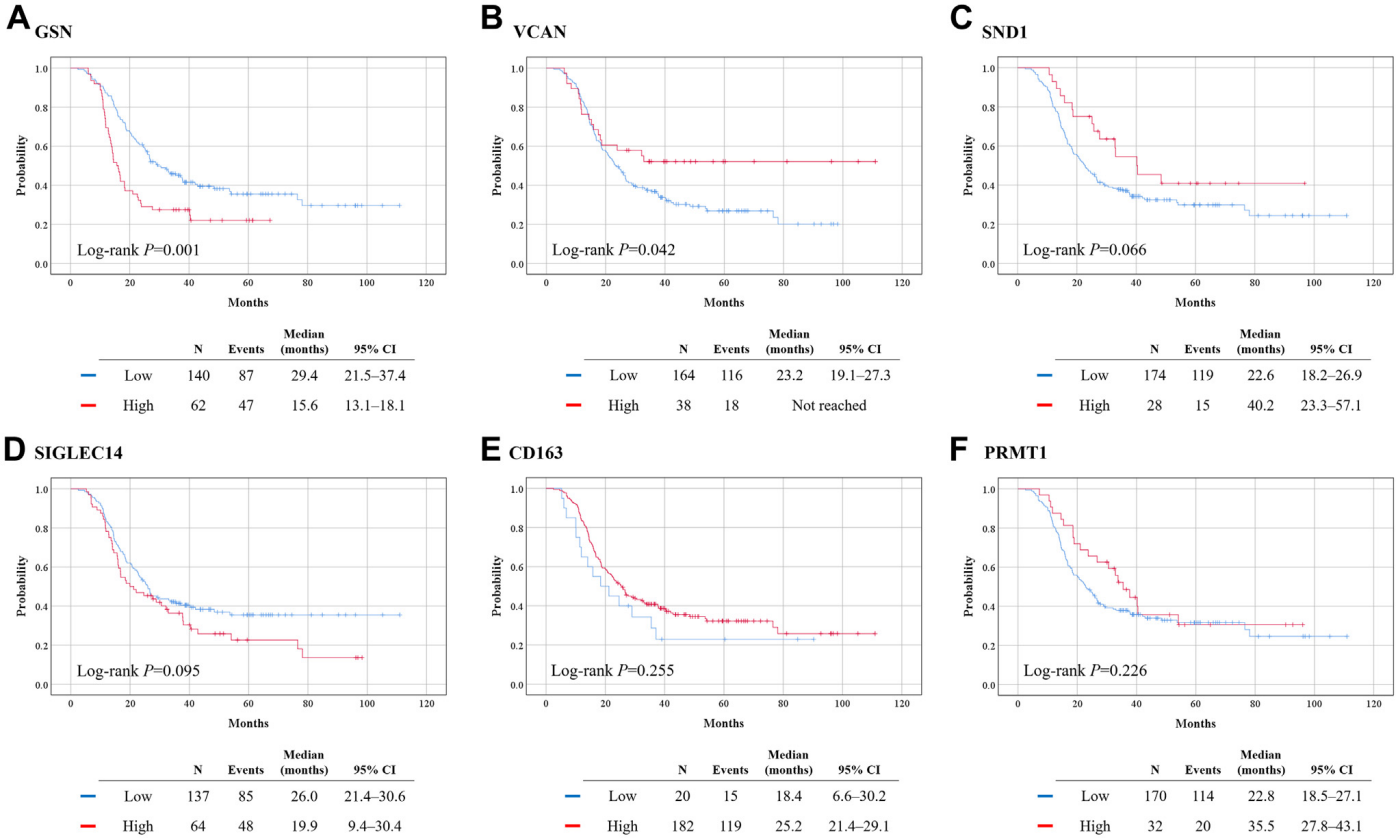
**Comparison of progression-free survival based on the plasma levels of proteins.***A*, GSN; *B*, VCAN; *C*, SND1; *D*, SIGLEC14; *E*, CD163; *F*, PRMT1.

In survival analysis, the high GSN group showed significantly worse PFS than did the low GSN group (median, 15.6 *versus* 29.4 months; *p* = 0.001). In contrast, the high VCAN group showed significantly better PFS than did the low VCAN group (median, not reached *versus* 23.2 months; *p* = 0.042). PFS was also better in the high than in the low SND1 group, but the difference was not statistically significant (median, 40.2 *versus* 22.6 months; *p* = 0.066). No differences in PFS were observed between groups with high and with low plasma levels of SIGLEC14, CD163, and PRMT1 (Fig. 3).

In the multivariate analysis adjusted for patient age, FIGO stage, and residual tumor after surgery, a high plasma GSN level was identified as an independent poor prognostic biomarker for PFS (aHR, 1.556; 95% CI, 1.073–2.256; *p* = 0.020). However, subsequent multivariate analyses revealed no influence of VCAN (aHR, 0.617; 95% CI 0.370–1.030; *p* = 0.065) and SND1 (aHR, 0.789; 95% CI 0.454–1.372; *p* = 0.401) plasma levels on PFS (Table 3).

**Table 3:** Factors associated with progression-free survival

Characteristics	*Univariate analysis*			*Multivariate Analysis*								
	HR	95% CI	*P*	aHR	95% CI	*P*	aHR	95% CI	*P*	aHR	95% CI	*P*
Age, years												
<55	Ref.			Ref.			Ref.			Ref.		
≥55	1.253	0.892–1.761	0.194	1.064	0.754–1.503	0.723	1.181	0.829–1.682	0.357	1.087	0.770–1.534	0.637
FIGO stage												
I-II	Ref.			Ref.			Ref.			Ref.		
III	2.644	1.281–5.457	0.009	2.151	1.027–4.506	0.042	2.022	0.961–4.253	0.063	2.112	1.006–4.433	0.048
IV	4.440	2.064–9.551	<0.001	3.177	1.442–7.000	0.004	3.208	1.454–7.076	0.004	3.283	1.484–7.264	0.003
Residual tumor after surgery												
No gross	Ref.			Ref.			Ref.			Ref.		
Gross	2.313	1.617–3.308	<0.001	1.898	1.311–2.747	0.001	1.954	1.355–2.817	<0.001	1.949	1.349–2.816	<0.001
GSN												
Low	Ref.			Ref.								
High	1.850	1.292–2.648	0.001	1.556	1.073–2.256	0.020						
VCAN												
Low	Ref.						Ref.					
High	0.601	0.365–0.988	0.045				0.617	0.370–1.030	0.065			
SND1												
Low	Ref.									Ref.		
High	0.607	0.355–1.040	0.069							0.789	0.454–1.372	0.401

Abbreviations: aHR, adjusted hazard ratio; CA-125, cancer antigen 125; CI, confidence interval; FIGO, International Federation of Gynecology and Obstetrics; HR, hazard ratio; Ref., reference.

### Development of Models Predicting 18-Month PFS Rate

**Fig. 4:**
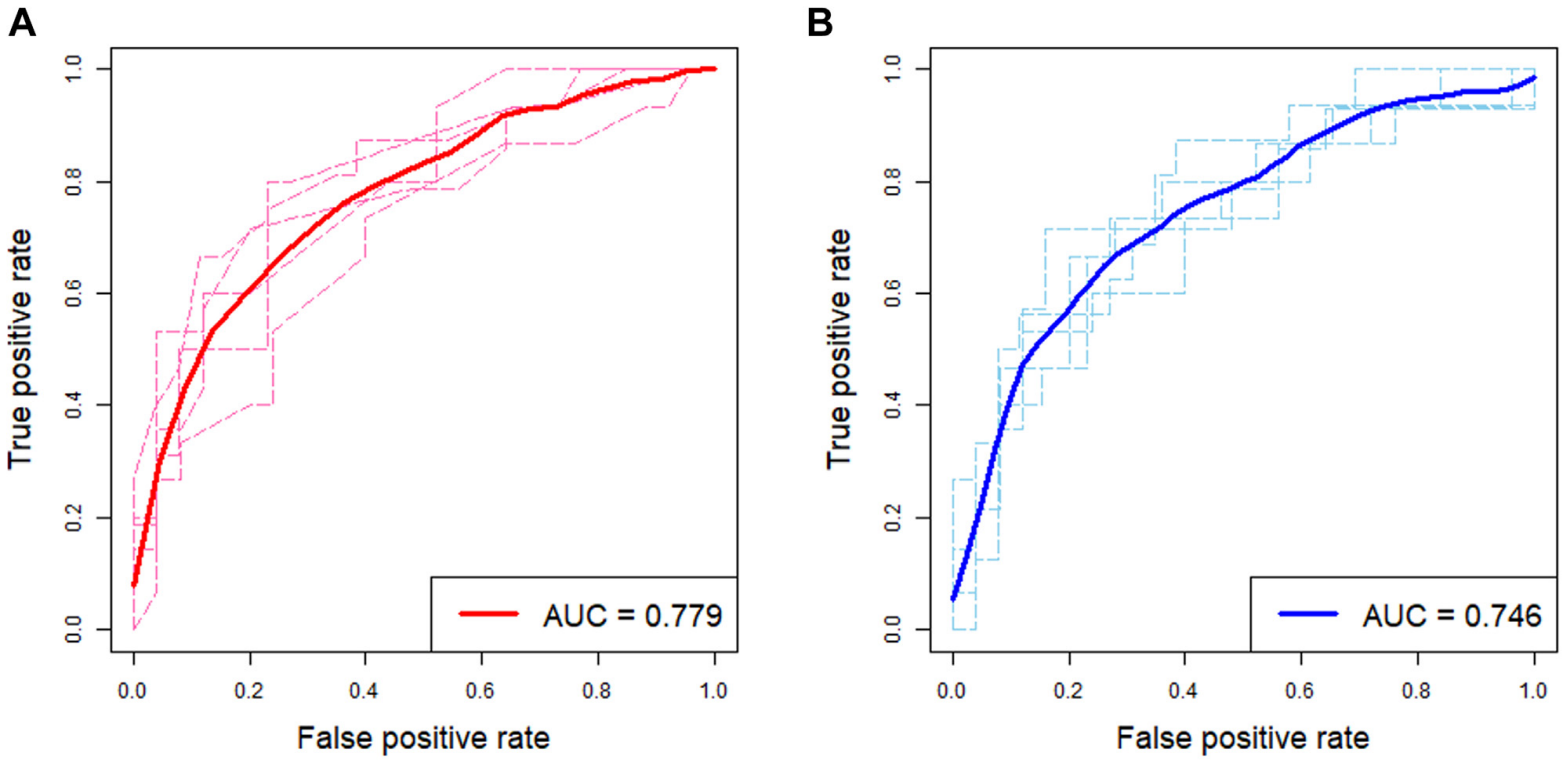
**Predictive performance of the developed models.** Receiver operating characteristic curves with the areas under the receiver operating characteristic curves (AUCs) for 18-month progression-free survival rate. The regression-based models underwent 5-fold cross-validation. *A*, a model using the cutoff values for plasma GSN and VCAN; *B*, a model using the continuous values for plasma GSN and VCAN.

Next, we constructed regression-based models predicting the 18-month PFS rate using clinical variables and plasma levels of five plasma protein biomarkers in patients with HGSOC (n = 202). Herein, SND1 was excluded as the high-SND1 group showed better PFS than did the low-SND1 group in the validation phase, which was contrary to the results in the development phase. Through stepwise selection methods, four predictors were selected: FIGO stage, residual tumor after surgery, GSN, and VCAN. Various models were developed using these predictors. Each predictive model underwent 5-fold cross-validation to compute the AUC. Among them, the model using cutoff plasma values for GSN (24.350 ng/ml) and VCAN (5.832 ng/ml) showed the best predictive performance, with an AUC of 0.779 (Fig. 4 and supplemental Table S9). This model also showed better predictive performance than did the model using continuous values for plasma GSN and VCAN levels, and those replacing the two protein biomarkers, GSN and VCAN, with serum CA-125 levels (supplemental Table S9).

**Fig. 5:**
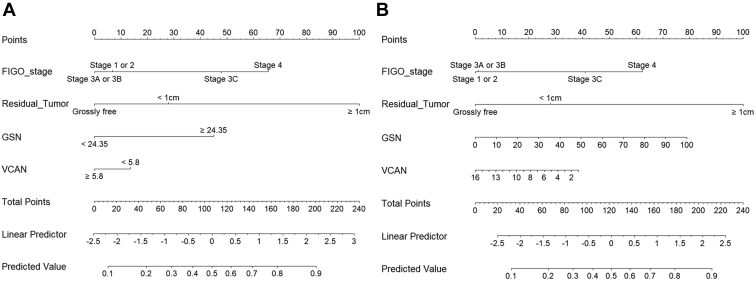
**Regression-based nomograms predicting 18-month progression-free survival rate.***A*, a nomogram using the cutoff values for plasma GSN and VCAN; *B*, a nomogram using the continuous values for plasma GSN and VCAN.

Using regression-based models, nomograms were then developed for clinical use (Fig. 5). Finally, we fitted a user-friendly interface onto the developed nomograms and posted them on a website (http://asiansgo.org/software/nomogram_ovarian).

## Discussion

Our proteomic analysis study identified plasma protein biomarkers that might be associated with the prognosis of HGSOC. In validation with ELISA, high plasma levels of GSN were associated with worse PFS, while VCAN, SND1, SIGLEC14, CD163, and PRMT1 did not affect the survival outcomes of patients with HGSOC. We also developed models and nomograms to predict the 18-month PFS rate for clinical purposes.

GSN, a calcium-dependent multifunctional actin-binding protein, has cytoplasmic and plasma isoforms, which are encoded by the same gene (31). Plasma GSN is a well-known poor prognostic biomarker for PFS and OS in patients with ovarian cancer. In addition, the expression and secretion of GSN were higher in chemoresistant ovarian cancer cells than in chemosensitive ovarian cancer cells (32). Consistently, the current study showed that high plasma GSN levels were associated with poor prognostic factors, such as advanced-stage disease and residual tumor after surgery, loss of platinum sensitivity, and reduced PFS. Recently, Asare–Werehene *et al*. demonstrated that plasma GSN confers chemoresistance in ovarian cancer by inhibiting the antitumor functions of macrophages through apoptosis and modulating the tumor microenvironment (33).

VCAN, a large extracellular matrix proteoglycan, is known to play role in promoting tumorigenesis and enhancing tumor progression and metastasis (34). Researchers have reported positive associations between high tissue expression of VCAN and poor survival outcomes in various malignancies including breast cancer (35) and renal cell carcinoma (36). In advanced-stage serous ovarian cancer, Ghosh *et al*. reported that high VCAN expression in the tumor stroma was associated with increased angiogenesis and significantly worse PFS and OS than low VCAN expression (37). However, such an association seems to differ depending on the specimen type. In contrast to this study, we measured plasma VCAN levels instead of tissue expression and observed that VCAN did not affect PFS in patients with HGSOC.

SND1, a component of the RNA-induced silencing complex, is an oncogene involved in tumorigenesis, tumor progression, and metastasis in multiple malignancies, including breast cancer (38) and colorectal cancer (39). In ovarian cancer, SND1 promotes epithelial-to-mesenchymal transition, which facilitates metastasis of ovarian cancer (40). Furthermore, Wang *et al*. reported that miR-1224-5p inhibits the proliferation and invasion of ovarian cancer by targeting SND1 (41). Recently, Cui *et al*. suggested a potential correlation between the tissue expression of SND1 and tumor mutational burden or microsatellite instability across all The Cancer Genome Atlas tumors (42). In contrast, our study showed that high or low plasma SND1 levels did not affect PFS in patients with HGSOC. Such inconsistent results between our study and previous studies might originate from differences in specimen types, histological subtypes, and sample sizes. To the best of our knowledge, no previous study has investigated the relationship between plasma SND1 levels and survival outcomes in ovarian cancer. Therefore, further prospective studies are warranted to investigate the relationship between plasma SND1 levels and survival outcomes.

CD163, a multifunctional receptor containing a scavenger receptor cysteine-rich domain, is specifically expressed in monocytes and macrophages and can be cleaved from the cell membrane of monocytes and macrophages (43). Besides its multiple functions, such as immune modulation, high serum CD163 levels have been associated with poor survival outcomes in various malignancies (44, 45, 46), including ovarian cancer. No *et al*. reported that high serum CD163 levels were an independent poor prognostic factor for PFS in patients with epithelial ovarian cancer (n = 55) (47). In contrast, no reduction in PFS due to high plasma CD163 levels was observed in our study. While a previous study examined serum samples of patients with all histological subtypes and grades of epithelial ovarian cancer, the current study examined plasma samples of patients with HGSOC. Such differences may underlie the inconsistent results.

PRMT1 mediates epigenetic modifications. Aberrant expression of PRMT1 has been reported to be involved in tumorigenesis (48) and is an unfavorable prognostic biomarker in breast cancer (49) and colorectal cancer (50). In non–small cell lung cancer, PRMT1 has been suggested to be a regulator of epithelial-to-mesenchymal transition (51). Recently, Matsubara *et al*. investigated the prognostic role of PRMT1 tissue expression in patients with ovarian serous carcinoma (n = 51) (52). They found that high PRMT1 expression was associated with platinum resistance and reduced OS. In contrast, we could not identify any association between plasma PRMT1 levels and response to platinum-based chemotherapy or PFS.

SIGLEC family proteins play diverse immune and nonimmune regulatory roles in the tumor microenvironment and participate in tumor progression. Facilitating tumor immune escape is one of the mechanisms by which tumors progress (53). Compared with other SGILEC family proteins, the prognostic role of SIGLEC14 in ovarian cancer is not fully understood. We observed no association between plasma SIGLEC14 levels and PFS in patients with HGSOC.

In the current study, we developed two regression-based models and nomograms to predict the 18-month PFS rate in patients with newly diagnosed HGSOC. In both models, only two (GSN and VCAN) of the six protein biomarkers were selected and incorporated. Although independent multivariate analyses indicated GSN as the solitary independent prognostic factor for PFS, the addition of VCAN to GSN seems to confer further improvement in performance in predicting the 18-month PFS rate. In the validation phase, we could not conduct external validation due to the scarcity of resources and time to conduct a prospective multicenter study that could collect plasma samples from the enrolled subjects. Instead, we implemented 5-fold cross-validation, a well-established statistical method, to prevent overfitting and increase the robustness and prediction accuracy of the developed model. A further increase in the predictive performance is expected if the multiomics data of patients with HGSOC are integrated into the developed models.

Throughout the study, patients’ *BRCA1/2* mutational status and the use of first-line PARP inhibitor maintenance treatment were not considered, because of their low frequency in our study. In particular, only a few patients were eligible (n = 3 in the second phase). Such a low frequency might have originated from the sociomedical environment in Korea. In October 2019 and August 2020, the Korean Ministry of Food and Drug Safety approved olaparib and niraparib as first-line maintenance therapies based on the SOLO1 (6) and PRIMA trials (7), respectively. Furthermore, it was not until October 2021 that the National Health Insurance System started to cover both olaparib and niraparib in patients with *BRCA1/2* mutated HGSOC. Despite the approval of PARP inhibitors, patients with HGSOC find these difficult to use beyond insurance coverage because of their high cost.

In the era of precision cancer medicine, it is critical to predict prognosis or survival outcomes precisely. Our results indicated that adding plasma levels of GSN and VCAN to the clinical factors in predictive models improved the models’ performance. Applying the developed models, if a patient with HGSOC was predicted to be at high risk of disease progression within 18 months from the initiation of primary treatment, physicians might consider incorporating bevacizumab into conventional taxane- and platinum-based chemotherapy. In particular, based on the germline/somatic *BRCA1/2* mutational status and homologous recombination deficiency, first-line PARP inhibitor maintenance therapy may be recommended more strongly (54). After completion of chemotherapy, a high-risk patient might undergo more intensive surveillance.

Our study had several limitations. First, as this study had a retrospective design, inevitable issues, such as selection bias, might exist. Second, the sample size might have been insufficient for discovering and validating plasma protein biomarkers. In particular, in the validation phase, we failed to observe a relationship of high plasma SND1 levels with poor prognosis, which was marked in the development phase. Third, external validation of the developed models is needed. Fourth, we only investigated statistical correlations but did not evaluate the biological interactions between protein biomarkers. Lastly, we did not investigate longitudinal changes in each plasma protein biomarker over the course of the primary treatment. Such information might enable us to calculate the kinetics of each biomarker during a specific period and predict the primary treatment success more accurately.

In conclusion, we successfully generated proteomic profiles of plasma samples from patients with HGSOC. A subsequent ELISA study assessed the prognostic value of the six protein biomarkers. Plasma GSN was identified as a poor prognostic biomarker for PFS in HGSOC, but plasma VCAN, SND1, SIGLEC14, CD163, and PRMT1 levels were not. Combined with clinical factors, we developed models and nomograms to predict the 18-month PFS rate for clinical purposes. Our study results provided insights into the protein biomarkers that might potentially develop HGSOC and offered clues for developing therapeutic targets. Further translational and prospective validation studies are needed.

## Data Availability

The mass spectrometry proteomics data have been deposited to the ProteomeXchange Consortium (http://proteomecentralproteomexchange.org) *via* the PRIDE partner repository with the dataset identifier PXD034636.

## Supplemental data

This article contains supplemental data.

## Conflict of Interest

The authors declare no competing interests.
