# Supplementary material for: Proteomic Discovery of Plasma Protein Biomarkers and Development of Models Predicting Prognosis of High-Grade Serous Ovarian Carcinoma
Source: Mol Cell Proteomics. 2023 Jan 17;22(3):100502. doi: 10.1016/j.mcpro.2023.100502 (PMC9972571; doi:10.1016/j.mcpro.2023.100502)
Supplement: Supplemental table 2 [file mmc2.docx]

**Supplementary Table 2.** Comparisons of clincopathologic characteristics between good and poor prognosis groups in discovery phase

| **Characteristics** | **All**  **(n=20, %)** | **Good prognosis**  **(n=10, %)** | **Poor prognosis**  **(n=10, %)** | ***P*** |
| --- | --- | --- | --- | --- |
| Age, years |  |  |  |  |
| Mean ± SD | 54.9 ± 8.9 | 53.9 ± 7.1 | 56.0 ± 10.7 | 0.609 |
| Parity |  |  |  |  |
| Median (range) | 2 (0–5) | 2 (0–4) | 2 (1–5) | 0.594 |
| Menopausal status |  |  |  |  |
| Menopause | 14 (70.0) | 8 (80.0) | 6 (60.0) | 0.628 |
| Serum CA-125, IU/mL |  |  |  |  |
| Median (range) | 976.5 (32.6–9098.0) | 556.5 (32.6–3545.0) | 1218.5 (134.8–9098.0) | 0.364 |
| FIGO stage |  |  |  | >0.999 |
| III | 15 (75.0) | 8 (80.0) | 7 (70.0) |  |
| IV | 5 (25.0) | 2 (20.0) | 3 (30.0) |  |
| Residual tumor after PDS |  |  |  | >0.999 |
| No gross | 14 (70.0) | 7 (70.0) | 7 (70.0) |  |
| <1 cm | 6 (30.0) | 3 (30.0) | 3 (30.0) |  |
| Total cycles of POAC |  |  |  | >0.999 |
| 6 | 16 (80.0) | 8 (80.0) | 8 (80.0) |  |
| 9 | 4 (20.0) | 2 (20.0) | 2 (20.0) |  |
| Recurrence |  |  |  |  |
| No | 5 (25.0) | 5 (50.0) | 0 | 0.033 |
| Yes | 15 (75.0) | 5 (50.0) | 10 (100.0) |  |
| PSR^a^ | 11 (55.0) | 5 (50.0) | 6 (60.0) | 0.231 |
| PRR | 4 (20.0) | 0 | 4 (40.0) |  |
| Platinum sensitivity |  |  |  | 0.087 |
| Platinum-sensitive^b^ | 16 (80.0) | 10 (100.0) | 6 (60.0) |  |
| Platinum-resistant | 4 (20.0) | 0 | 4 (40.0) |  |
| g*BRCA* mutational status^c^ |  |  |  | 0.349 |
| Both wild-type | 11 (55.0) | 4 (40.0) | 7 (70.0) |  |
| *BRCA1* mutation | 7 (35.0) | 5 (50.0) | 2 (20.0) |  |
| *BRCA2* mutation | 2 (10.0) | 1 (10.0) | 1 (10.0) |  |
| Abbreviations: CA-125, cancer antigen 125; FIGO, International Federation of Gynecology and Obstetrics; PDS, primary debulking surgery; POAC, post-operative adjuvant chemotherapy; PRR, platinum-resistant recurrence; PSR, platinum-sensitive recurrence; SD, standard deviation.  ^a^PSR was defined as relapse ≥6 months after completion of taxane- and platinum-based chemotherapy, whereas PRR as relapse <6 months.  ^b^In addition to PSR, the patients who completed taxane- and platinum-based chemotherapy and did not experience disease recurrence during at least six months of follow-up period were considered platinum-sensitive.  ^c^Germline *BRCA1/2* mutational status. | | | | |
