# Supplementary material for: Proteomic Discovery of Plasma Protein Biomarkers and Development of Models Predicting Prognosis of High-Grade Serous Ovarian Carcinoma
Source: Mol Cell Proteomics. 2023 Jan 17;22(3):100502. doi: 10.1016/j.mcpro.2023.100502 (PMC9972571; doi:10.1016/j.mcpro.2023.100502)
Supplement: Supplemental table 7 [file mmc7.docx]

**Supplementary Table 7.** Correlations between serum CA-125 levels and plasma levels of protein biomarkers

|  | GSN,  ng/mL | VCAN,  ng/mL | SND1,  ng/mL | SIGLEC14^a^,  ng/mL | CD163,  ug/uL | PRMT1,  ng/mL |
| --- | --- | --- | --- | --- | --- | --- |
| CA-125, IU/mL |  |  |  |  |  |  |
| Pearson *r* | 0.118 | 0.051 | -0.008 | -0.021 | 0.077 | -0.072 |
| *P* | 0.093 | 0.471 | 0.913 | 0.763 | 0.276 | 0.307 |
| GSN, ng/mL |  |  |  |  |  |  |
| Pearson *r* |  | 0.224 | 0.177 | -0.009 | 0.351 | -0.015 |
| *P* |  | 0.001 | 0.012 | 0.899 | <0.001 | 0.829 |
| VCAN, ng/mL |  |  |  |  |  |  |
| Pearson *r* |  |  | 0.167 | 0.501 | 0.341 | -0.106 |
| *P* |  |  | 0.017 | <0.001 | <0.001 | 0.135 |
| SND1, ng/mL |  |  |  |  |  |  |
| Pearson *r* |  |  |  | 0.074 | 0.081 | 0.007 |
| *P* |  |  |  | 0.297 | 0.253 | 0.920 |
| SIGLEC14^a^, ng/mL |  |  |  |  |  |  |
| Pearson *r* |  |  |  |  | -0.017 | -0.027 |
| *P* |  |  |  |  | 0.806 | 0.704 |
| CD163, ug/uL |  |  |  |  |  |  |
| Pearson *r* |  |  |  |  |  | 0.003 |
| *P* |  |  |  |  |  | 0.966 |
| Missing data: ^a^1. | | | | | | |
