# Supplementary material for: Proteomic Discovery of Plasma Protein Biomarkers and Development of Models Predicting Prognosis of High-Grade Serous Ovarian Carcinoma
Source: Mol Cell Proteomics. 2023 Jan 17;22(3):100502. doi: 10.1016/j.mcpro.2023.100502 (PMC9972571; doi:10.1016/j.mcpro.2023.100502)
Supplement: Supplemental table 8 [file mmc8.docx]

**Supplementary Table 8.** Comparisons of patients’ clinicopathologic characteristics by ELISA results

|  | **GSN** | | | **VCAN** | | | **SND1** | | | **SIGLEC14** | | | **CD163** | | | **PRMT1** | | |
| --- | --- | --- | --- | --- | --- | --- | --- | --- | --- | --- | --- | --- | --- | --- | --- | --- | --- | --- |
|  | **Low**  **(n=140)** | **High**  **(n=62)** | ***P*** | **Low**  **(n=164)** | **High**  **(n=38)** | ***P*** | **Low**  **(n=174)** | **High**  **(n=28)** | ***P*** | **Low**  **(n=137)** | **High**  **(n=64)** | ***P*** | **Low**  **(n=20)** | **High**  **(n=182)** | ***P*** | **Low**  **(n=170)** | **High**  **(n=32)** | ***P*** |
| Age, years |  |  |  |  |  |  |  |  |  |  |  |  |  |  |  |  |  |  |
| Mean | 55.4 | 60.7 | 0.001 | 55.4 | 64.2 | <0.001 | 56.8 | 58.2 | 0.341 | 56.9 | 57.4 | 0.749 | 54.8 | 57.3 | 0.331 | 57.0 | 57.2 | 0.915 |
| CA-125, IU/mL |  |  |  |  |  |  |  |  |  |  |  |  |  |  |  |  |  |  |
| Median | 654.9 | 1389.5 | 0.043 | 813.5 | 758.5 | 0.884 | 820.0 | 542.5 | 0.702 | 871.0 | 646.3 | 0.671 | 372.8 | 864.0 | 0.143 | 820.0 | 709.0 | 0.814 |
| FIGO stage |  |  | 0.012 |  |  | 0.505 |  |  | 0.032 |  |  | 0.687 |  |  | 0.824 |  |  | 0.389 |
| I-II | 20 (14.3) | 3 (4.8) |  | 17 (10.4) | 6 (15.8) |  | 17 (9.8) | 6 (21.4) |  | 17 (12.4) | 6 (9.4) |  | 3 (15.0) | 20 (11.0) |  | 21 (12.4) | 2 (6.3) |  |
| III | 94 (67.1) | 37 (59.7) |  | 106 (64.6) | 25 (65.8) |  | 111 (63.8) | 20 (71.4) |  | 86 (62.8) | 44 (68.8) |  | 13 (65.0) | 118 (64.8) |  | 107 (62.9) | 24 (75.0) |  |
| IV | 26 (18.6) | 22 (35.5) |  | 41 (25.0) | 7 (18.4) |  | 46 (26.4) | 2 (7.1) |  | 34 (24.8) | 14 (21.9) |  | 4 (20.0) | 44 (24.2) |  | 42 (24.7) | 6 (18.8) |  |
| Residual tumor after surgery |  |  | 0.011 |  |  | 0.772 |  |  | 0.027 |  |  | 0.284 |  |  | 0.217 |  |  | 0.084 |
| No gross | 108 (77.1) | 37 (59.7) |  | 117 (71.3) | 28 (73.7) |  | 120 (69.0) | 25 (89.3) |  | 102 (74.5) | 43 (67.2) |  | 12 (60.0) | 133 (73.1) |  | 118 (69.4) | 27 (84.4) |  |
| Gross | 32 (22.9) | 25 (40.3) |  | 47 (28.7) | 10 (26.3) |  | 54 (31.0) | 3 (10.7) |  | 35 (25.5) | 21 (32.8) |  | 8 (40.0) | 49 (26.9) |  | 52 (30.6) | 5 (15.6) |  |
| Platinum sensitivity |  |  | 0.040 |  |  | 0.233 |  |  | 0.087 |  |  | 0.538 |  |  | 0.088 |  |  | 0.427 |
| Sensitive | 124 (88.6) | 48 (77.4) |  | 142 (86.6) | 30 (78.9) |  | 145 (83.3) | 27 (96.4) |  | 118 (86.1) | 53 (82.8) |  | 14 (70.0) | 158 (86.8) |  | 143 (84.1) | 29 (90.6) |  |
| Resistant | 16 (11.4) | 14 (22.6) |  | 22 (13.4) | 8 (21.1) |  | 29 (16.7) | 1 (3.6) |  | 19 (13.9) | 11 (17.2) |  | 6 (30.0) | 24 (13.2) |  | 27 (15.9) | 3 (9.4) |  |
| Abbreviations: CA-125, cancer antigen 125; FIGO, International Federation of Gynecology and Obstetrics; SD, standard deviation. | | | | | | | | | | | | | | | | | | |
