# Supplementary material for: Proteomic Discovery of Plasma Protein Biomarkers and Development of Models Predicting Prognosis of High-Grade Serous Ovarian Carcinoma
Source: Mol Cell Proteomics. 2023 Jan 17;22(3):100502. doi: 10.1016/j.mcpro.2023.100502 (PMC9972571; doi:10.1016/j.mcpro.2023.100502)
Supplement: Supplemental table 9 [file mmc9.docx]

**Supplementary Table 9.** Developed models predicting 18-month PFS rate in high-grade serous ovarian carcinoma

| **Model** | ***Training*** | | | | ***Validation*** | | | |
| --- | --- | --- | --- | --- | --- | --- | --- | --- |
|  | **AUC** | **Sensitivity** | **Specificity** | **Balanced accuracy** | **AUC** | **Sensitivity** | **Specificity** | **Balanced accuracy** |
| FIGO stage, residual tumor after surgery, GSN (cut-off, 24.350 ng/mL), VCAN (cut-off, 5.832 ng/mL) | 0.783 | 0.720 | 0.720 | 0.720 | 0.779 | 0.703 | 0.725 | 0.714 |
| FIGO stage, residual tumor after surgery, GSN (continuous), VCAN (continuous) | 0.771 | 0.710 | 0.722 | 0.716 | 0.746 | 0.692 | 0.709 | 0.701 |
| FIGO stage, residual tumor after surgery, CA-125 (continuous) | 0.760 | 0.767 | 0.589 | 0.678 | 0.745 | 0.785 | 0.559 | 0.672 |
| Abbreviations: AUC, area under the receiver operating characteristic curve; CA-125, cancer antigen 125; FIGO, International Federation of Gynecology and Obstetrics. | | | | | | | | |
