# Supplementary figures and images for: Proteomic Discovery of Plasma Protein Biomarkers and Development of Models Predicting Prognosis of High-Grade Serous Ovarian Carcinoma
Source: Mol Cell Proteomics. 2023 Jan 17;22(3):100502. doi: 10.1016/j.mcpro.2023.100502 (PMC9972571; doi:10.1016/j.mcpro.2023.100502)

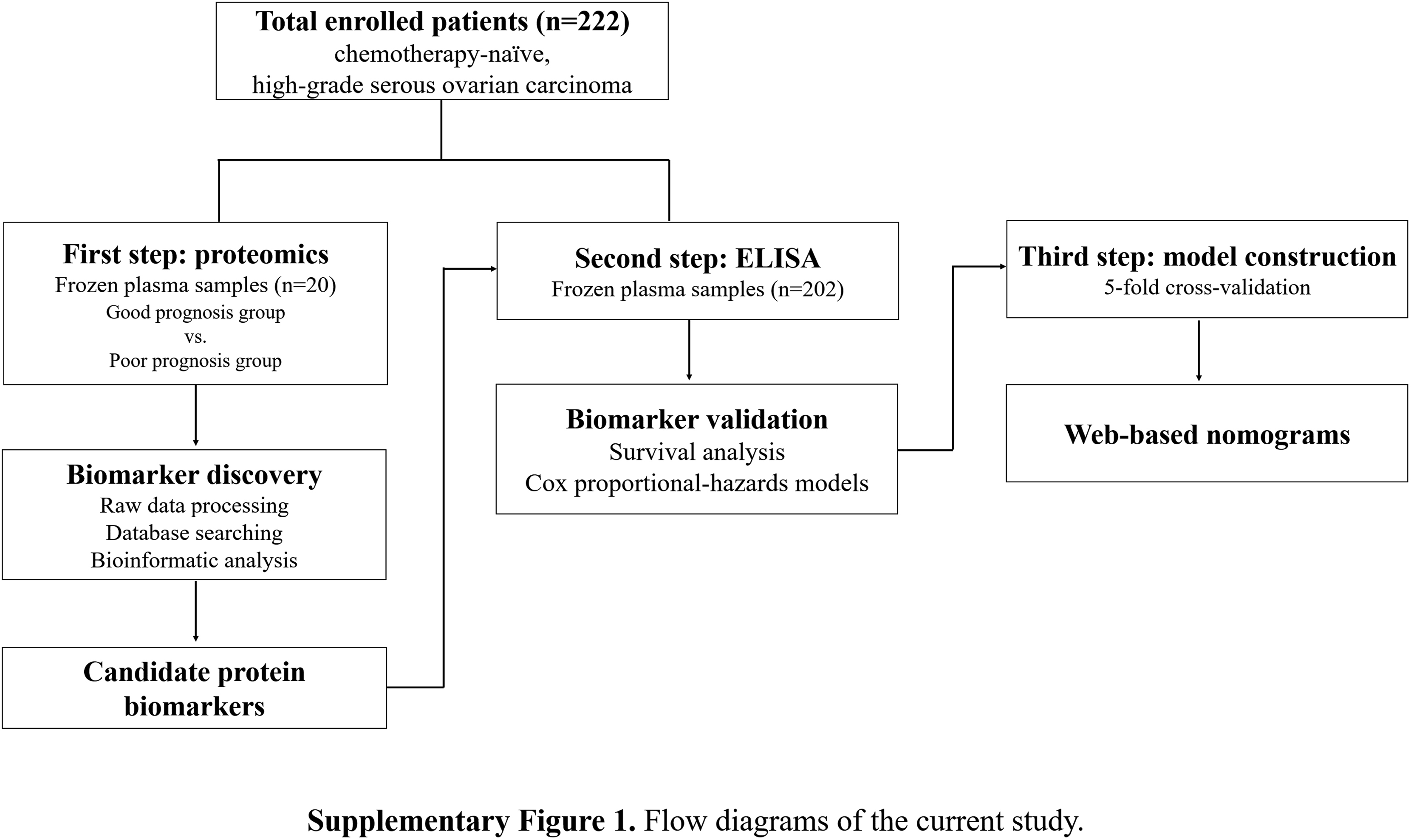

Supplement: Supplementary Figure 1 [file figs1.jpg]

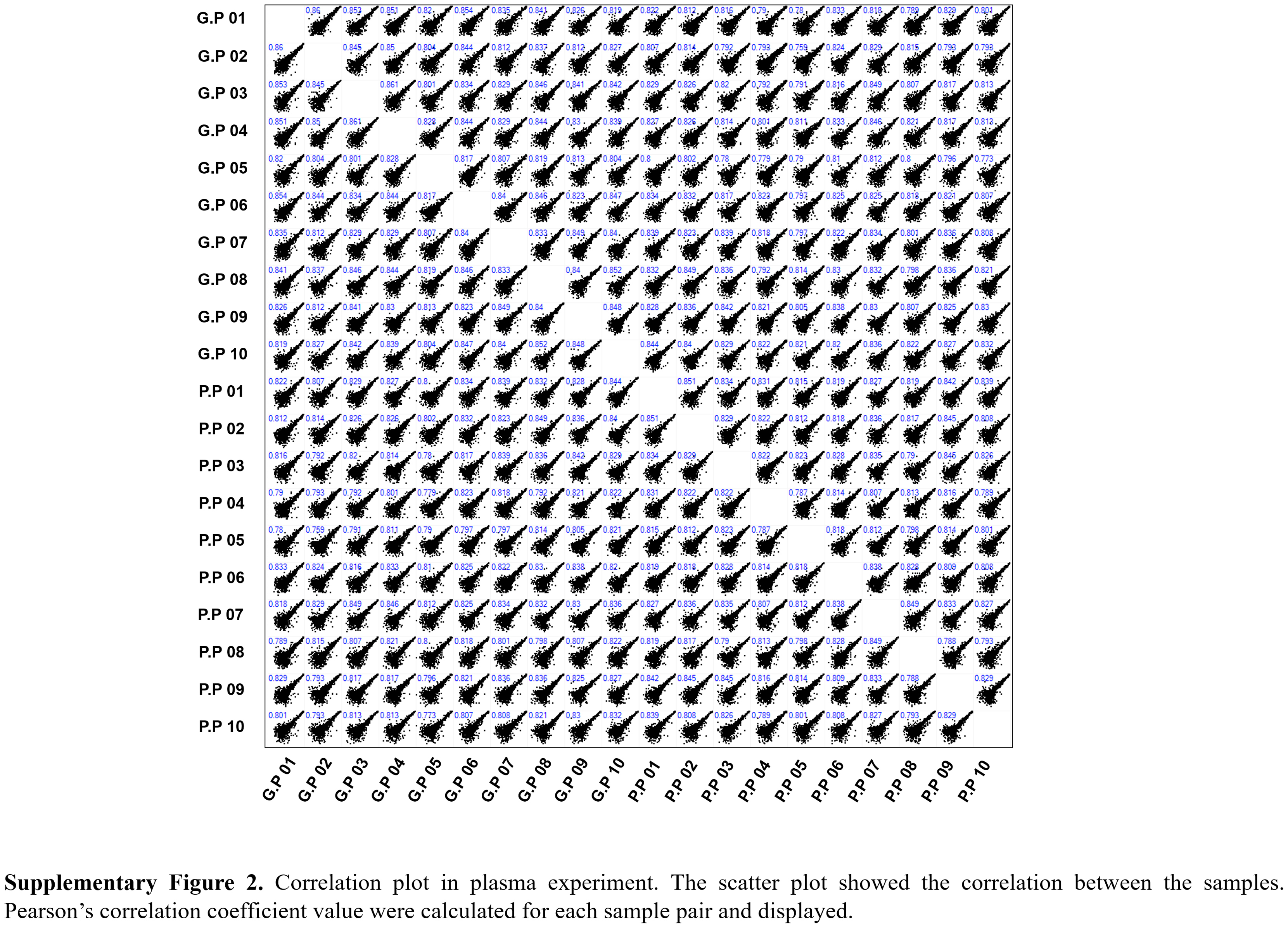

Supplement: Supplementary Figure 2 [file figs2.jpg]

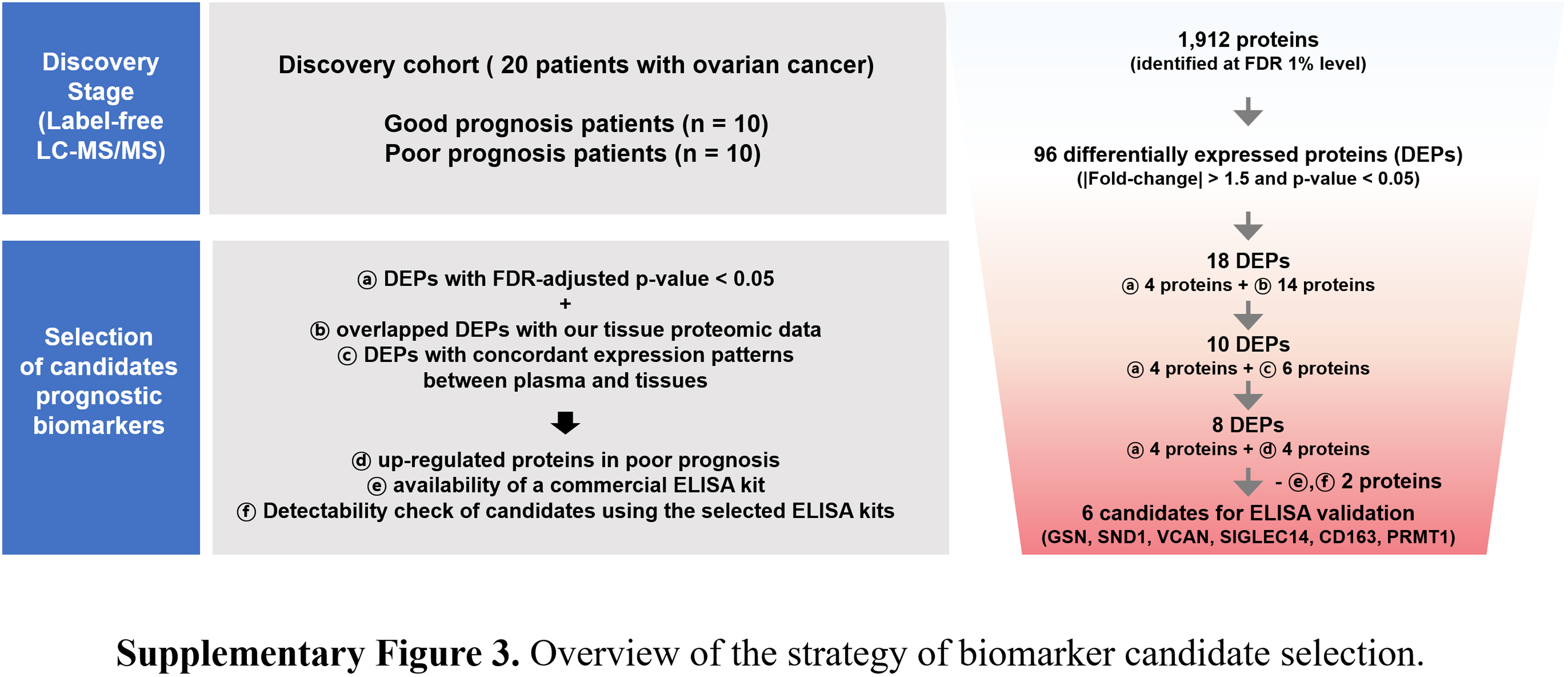

Supplement: Supplementary Figure 3 [file figs3.jpg]

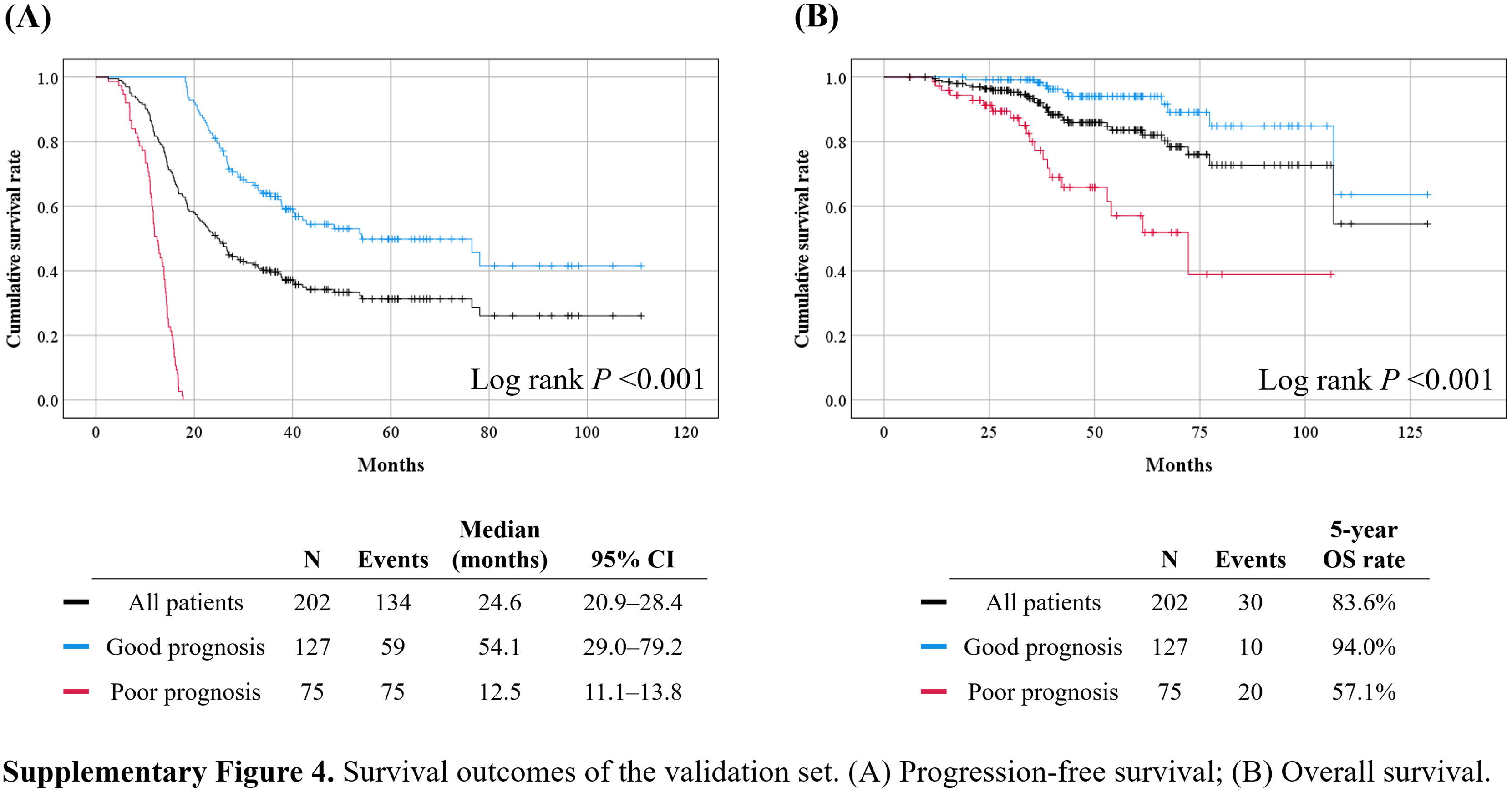

Supplement: Supplementary Figure 4 [file figs4.jpg]

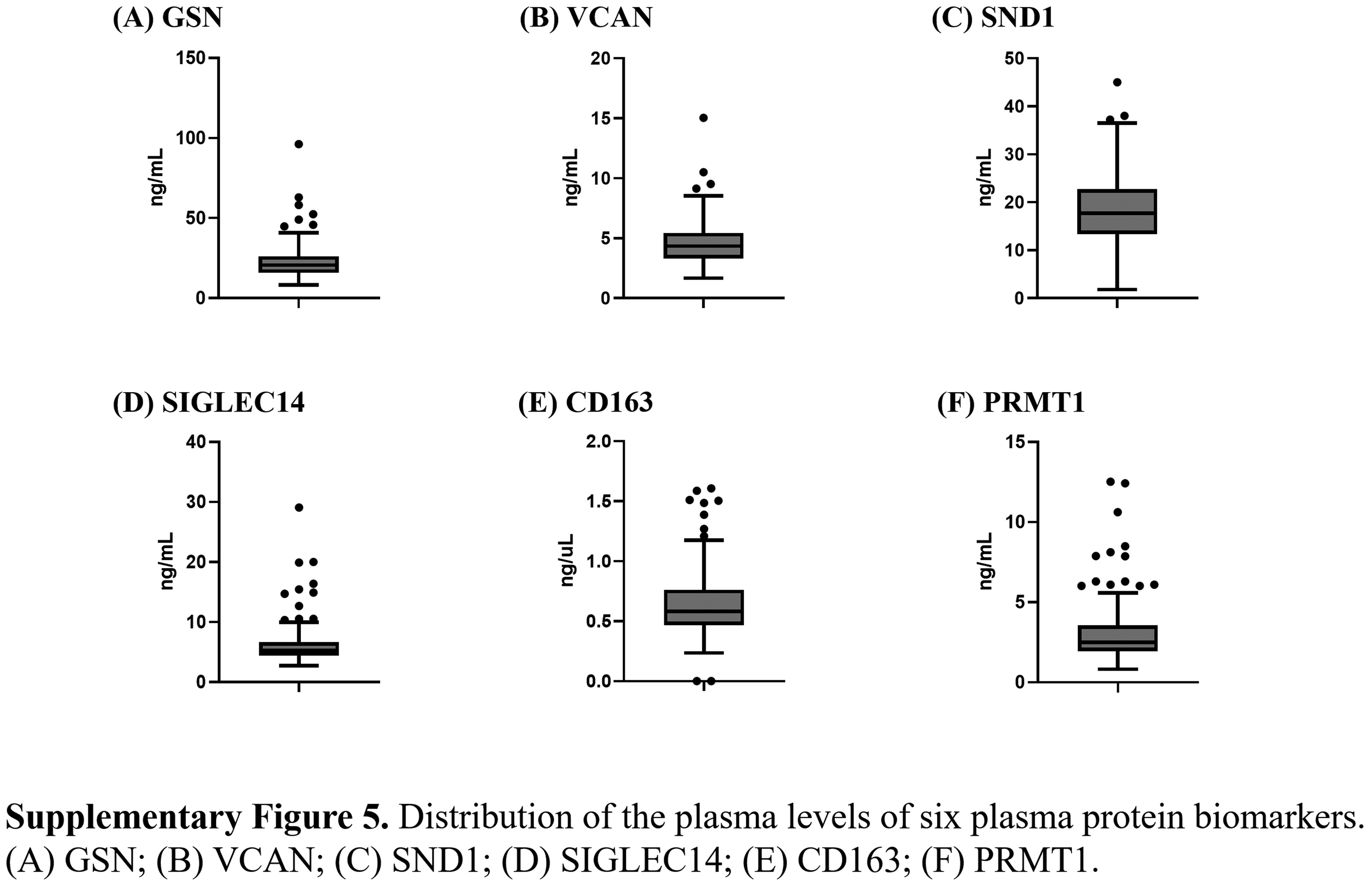

Supplement: Supplementary Figure 5 [file figs5.jpg]
